# Identification of crucial miRNAs and the targets in renal cortex of hypertensive patients by expression profiles

**DOI:** 10.1080/0886022X.2016.1244083

**Published:** 2016-11-02

**Authors:** Guohua Wang, Lan Wu, Zhi Chen, Jinghui Sun

**Affiliations:** a Department of Pediatrics, The First Hospital of Jilin University, Changchun, China;; b Department of Nephrology, The First Hospital of Jilin University, Changchun, China

**Keywords:** Hypertension, kidney, miRNA, microarray, protein–protein interaction

## Abstract

**Backgrounds:** Defect in kidney is one major reason of hypertension. The study aimed ao uncovering the regulatory mechanisms of miRNAs and the targets in hypertensive kidney.

**Methods:** Gene expression profile of GSE28345 and miRNA expression profile of GSE28283 were downloaded from GEO database. After data preprocessing, differently expressed genes (DEGs) and miRNAs (DE-miRs) were identified using limma package. Then targets of miRNAs were predicted according to information in relevant databases. Function and pathway enrichment analyses were performed for DEGs using DAVID software. Furthermore, protein–protein interaction (PPI) networks were constructed for up- and down-regulated genes, respectively, using the Cytoscape. Additionally, for down-regulated DEGs, the integrated regulatory network was established combining PPI network with the miRNA–mRNA interactions.

**Results:** As a result, 285 DEGs were identified, including 177 up-regulated and 108 down-regulated genes. Combined with the predicted targets of miRNAs, 22 up-regulated DE-miRs were identified. In the integrated network for down-regulated DEGs, three crucial nodes were identified as *ASPN*, *COL12A1,* and *SCN2A*. *ASPN* was predicted as target of miR-21 and miR-374b, and *COL12A1* was the target of miR-30e, miR-21, and miR-195, while *SCN2A* was the target of miR-30e, miR-374b, and miR-195. Notably, *COL12A1* and *ASPN* were linked with each other in the network.

**Conclusion:** Three crucial genes were identified in hypertensive kidney, such as *COL12A1*, *ASPN,* and *SCN2A*. *ASPN* might co-function with *COL12A1*, and they both might be the targets of miR-21. *SCN2A* might be a novel target of miR-30e and miR-374b. However, more experiments are needed to validate these results.

## Introduction

Hypertension is the major risk factor that leads to cardiovascular disease with high mortality and morbidity.^1^ It is often accompanied with numerous complications such as stroke, heart failure, and kidney diseases.^2^ In the United States, although the management and control of hypertension have been dramatically improved from 1999 to 2010, the prevalence remains as stable as 30% among these years.^3^

The endothelial dysfunction characterized by decreased synthesis of nitric oxide (NO) is a hallmark of hypertension. As the main extracellular cation, sodium retention in the extracellular could decrease the synthesis of NO. On the contrary, high plasma potassium, the main intracellular cation, has a beneficial effect on endothelial cells’ softness and could activate NO release.^4^ Therefore, it is recommended in the prevention of hypertension to keep the low-level of plasma sodium and high-level of potassium.^4^ It is theorized that hypertension is caused by a primary defect in the kidney.^5^ Kidney has a potent effect on the control of sodium excretion via the renin–angiotensin system (RAS), which was supposed to be the critical mechanism for blood pressure regulation.^6^ Therefore, it is urgent to investigate alterations in hypertensive kidneys.

Currently, various approaches including gene candidate analysis and genome wide search have been introduced into the identification of genetic components of hypertension,^7^ and several candidate genes were identified, such as *SPON1*, *GDF-15,* and *STK39.*^8–10^ However, gene alterations related to hypertensive kidney are rarely reported.

MicroRNAs (miRNAs) are small non-coding RNAs that could bind to complementary sequences of its targets and thereby result in the mRNAs’ degradation and the translation inhibition.^11^ They are key regulators for gene expressions. Reportedly, hcmv-miR-UL112 is associated with the increased risk of hypertension.^12^ Increased circulating miRNA-34a is detected in hypertensive patients with different liver diseases.^13^ Despite these profound findings, the molecular mechanisms of hypertension, especially kidney-related hypertension, remain obscure.

A recent study using microarray technology identifies differentially expressed genes (DEGs) and miRNA (DE-miRs) between kidneys of hypertensive patients and normotensive controls. As a result, the study finds 46 DEGs and 13 DE-miRs in the renal cortex between the two kinds of samples, and gene expressions, such as *APOE*, *SLC13A1,* and *CD36*; and miRNA expressions, such as hsa-miR-21, hsa-miR-181a, and hsa-miR-663 are validated.^14^ Nevertheless, the criteria for DEGs selection are not rigorous because the detailed fold change is not provided. In addition, interactions among the DEGs from protein level are not considered. Therefore, we re-analyzed their data by combining the gene expression profile GSE28345 with the miRNA expression profile GSE28283, to take full advantage of this microarray data and thereby uncover the potential gene interactions at protein level and the miRNA–mRNA regulations, thus to provide novel insights into the pathology of hypertension and potential biomarkers for the prognosis.

## Methods

### Microarray data

The gene expression profile GSE28345 and the miRNA expression profile GSE28283^14^ were all obtained from the Gene Expression Omnibus (GEO, http://www.ncbi.nlm.nih.gov/geo, Bethesda, MD) database. Samples of the two profiles were the same, and they were from the renal cortex tissues. There were a total of eight samples: five were from hypertensive male patients (hypertensive samples) and three from normotensive male patients (control samples). The profile of GSE28345 was based on the platform of GPL6244 [HuGene-1_0-st] Affymetrix Human Gene 1.0 ST Array, and GSE28283 based on the GPL10850 Agilent-021827 Human miRNA Microarray (V3) (miRBase release 12.0 miRNA ID version) (Agilent Technologies, Palo Alto, CA).

### Preprocessing of the two profiles and selection of DEG or DE-miRs

Raw data of the gene expression profile was normalized by Robust Multichip Average (RAM) algorithm,^15^ and the samples were divided into hypertensive group and control group. Then, Linear Models for Microarray Analysis (limma, http://www.bioconductor.org/packages/release/bioc/html/limma.html) of Bioconductor R^16^ was recruited to identify the differently expressed genes (DEGs). The thresholds were |log 2 fold change (FC)| >0.58 and *p* values <.05.

For miRNA profile, the raw data was interpreted by limma package to obtain signal intensity, and then were undergone the normexp background correction^17^ and quartile normalization.^18^ Thereafter, *t*-test in limma package was utilized to identify DE-miRs between two groups. The cutoff values were |log 2 FC| ≥ 1 and *p* values <.05.

### The prediction of target genes of miRNA

Databases including miRanda (http://mirdb.org/miRDB), PicTar (http://pictar.mdc-berlin.de), PITA,^19^ TargetScan (www.targetscan.org) and MirTarget2^20^ were searched to identify target genes of miRNAs. The interplayed miRNA–mRNAs that were recorded in at least three of the above databases were selected. Considering that miRNAs always regulate mRNA’s degradation and translation inhibition by binding to 3’ UTR of the mRNA, we extracted the interaction information between the corresponding transcripts (RefseqID) and miRNAs to reveal the interactions of miRNA–target.

### The correlation analysis between differently expressed mRNAs and miRNAs

In the identified transcript–miRNAs, the interactions with reverse expressions were screened out. Subsequently, the transcript RefseqID was converted into gene symbols.

### Functional enrichment analyses of DEGs

The Gene ontology (GO, http://www.geneontology.org/) and Kyoto Encyclopedia of Genes and Genomes (KEGG, http://www.genome.jp/kegg/pathway.html) pathway enrichment analyses were performed utilizing the Database for Annotation, Visualization and Integrated Discovery (DAVID, http://david.abcc.Ncifcrf.gov/) online software,^21^ to recognize potential biological processes and pathways that DEGs were involved in. The cutoff values for the significant function and pathways were *p* values <.05 and the count (gene number that enriched in a specific function or pathway term) ≥ 2.

### Construction of protein–protein interaction (PPI) network

By mapping the DEGs into Search Tool for the Retrieval of Interacting Genes (String, http://string-db.org/) database,^22^ the interaction relationships among DEGs at protein level were screened. Then, the protein–protein interaction (PPI) networks for up- and down-regulated DEGs were constructed under the criterion of combine score >0.4, respectively. The Cytoscape software was used to visualize the networks.^23^ We focused the down-regulated DEGs that might be down-regulated by the miRNAs, and the integrated regulatory network was constructed combining the PPI network of down-regulated genes with the miRNA–target interaction. The connectivity degrees were calculated through network statistical methods.

## Results

### Selection of DEGs and DE-miRs

According to the screening criteria, a total of 731 differently expressed transcripts were identified in the renal cortex tissues of hypertensive patients. Among them, 459 were up-regulated, corresponding to 177 DEGs; while 272 were down-regulated, corresponding to 108 DEGs.

Through the comparison of 859 known miRNAs between two groups, 22 up-regulated miRNAs in hypertensive group were identified. No down-regulated miRNAs were detected.

### The predicted target genes of miRNAs

Based on the interactions between miRNAs and the target transcripts, and the differently expressed mRNAs, the reversely expressed miRNA–transcript interactions were recognized. For all the identified miRNAs were up-regulated, only the down-regulated mRNAs were chosen to predict the miRNA–mRNA interaction. As a result, 23 transcripts were selected and converted into corresponding genes symbols, and the relevant miRNA–mRNA interactions were identified (Table 1).

**Table 1.** Regulatory relationship between miRNAs and differentially expressed genes.

microRNA	RefseqID	Gene symbol
hsa-miR-128	NM_001029854	*PDE8B*
hsa-miR-128, hsa-miR-132, hsa-miR-195, hsa-miR-30e	NM_153619	*SEMA6D*
hsa-miR-128	NM_153617	*SEMA6D*
hsa-miR-128, hsa-miR-132, hsa-miR-195, hsa-miR-30e	NM_153618	*SEMA6D*
hsa-miR-128, hsa-miR-342-3p	NM_172069	*PLEKHH2*
hsa-miR-128	NM_022842	*CDCP1*
hsa-miR-132, hsa-miR-223	NM_052832	*SLC26A7*
hsa-miR-132, hsa-miR-194, hsa-miR-195, hsa-miR-223, hsa-miR-30e, hsa-miR-374b	NM_001040143	*SCN2A*
hsa-miR-132, hsa-miR-215, hsa-miR-30e	NM_015236	*LPHN3*
hsa-miR-152	NM_000222	*KIT*
hsa-miR-152	NM_023037	*FRY*
hsa-miR-195, hsa-miR-21, hsa-miR-30e	NM_004370	*COL12A1*
hsa-miR-195	NM_014900	*COBLL1*
hsa-miR-21, hsa-miR-374b	NM_017680	*ASPN*
hsa-miR-223	NM_144710	*Sep10*
hsa-miR-28-5p	NM_032034	*SLC4A11*
hsa-miR-30e, hsa-miR-378	NM_020927	*VAT1L*
hsa-miR-30e, hsa-miR-374b	NM_021998	*ZNF711*
hsa-miR-342-3p	NM_030925	*CAB39L*
hsa-miR-374b	NM_005398	*PPP1R3C*
hsa-miR-374b	NM_002667	*PLN*
hsa-miR-374b	NM_006682	*FGL2*
hsa-miR-425	NM_002160	*TNC*

### The function and pathways of the DEGs

For the up-regulated DEGs, KEGG pathway analyses indicated that they were significantly enriched in four pathways such as arachidonic acid metabolism (e.g., *CYP2J2*, *CYP2B6,* and *GPX3*), metabolism of xenobiotics by cytochrome P450 (MOXBCP) (e.g., *UGT1A7*, *UGT1A10,* and *UGT1A6*), maturity onset diabetes of the young (e.g., *HNF1A*, *SLC2A2,* and *PKLR*) and Drug metabolism (DGM) pathways (e.g., *UGT1A7*, *UGT1A10,* and *UGT1A6*) (Table 2). Notably, genes enriched in MOXBCP and DGM pathways were identical. Meanwhile, GO analysis indicated that these up-regulated DEGs were mainly involved in transmembrane transport process and diverse metabolic processes of cofactor, triglyceride, acylglycerol, neutral lipid, and glycerol ether (Table 3).

**Table 2.** Pathways analyses for up- and down-regulated DEGs.

Term	Count	*p* Values	Genes
Up-regulated DEGs			
hsa00590:Arachidonic acid metabolism	4	0.029035948	*CYP2J2, CYP2B6, GPX3, GGT2*
hsa00980:Metabolism of xenobiotics by cytochrome P450	4	0.034662892	*(UGT1A7, UGT1A10, UGT1A6, UGT1A9, UGT1A8, UGT1A3, UGT1A5, UGT1A4, UGT1A1), UGT2A3, CYP2B6, MGST2*
hsa04950:Maturity onset diabetes of the young	3	0.035582143	*HNF1A, SLC2A2, PKLR*
hsa00982:Drug metabolism	4	0.037673733	*(UGT1A7, UGT1A10, UGT1A6, UGT1A9, UGT1A8, UGT1A3, UGT1A5, UGT1A4, UGT1A1), UGT2A3, CYP2B6, MGST2*
Down-regulated DEGs			
hsa00980:Metabolism of xenobiotics by cytochrome P450	3	0.042527459	*GSTM1, GSTM3, ADH1B*
hsa00982:Drug metabolism	3	0.045130713	*GSTM1, GSTM3, ADH1B*

DEG: differentially expressed genes.

**Table 3.** Biological processes for up- and down-regulated DEGs.

Term	Count	*p* Values
Up-regulated DEGs		
GO:0051186 cofactor metabolic process	10	6.28E–05
GO:0055085 transmembrane transport	15	5.55E–04
GO:0006641 triglyceride metabolic process	5	5.74E–04
GO:0006639 acylglycerol metabolic process	5	9.46E–04
GO:0006638 neutral lipid metabolic process	5	1.02E –03
GO:0006662 glycerol ether metabolic process	5	1.10E –03
GO:0018904 organic ether metabolic process	5	1.27E –03
GO:0006732 coenzyme metabolic process	7	2.50E–03
GO:0006979 response to oxidative stress	7	3.54E–03
Down-regulated DEGs		
GO:0035150 regulation of tube size	4	1.58E–03
GO:0050880 regulation of blood vessel size	4	1.58E–03
GO:0003018 vascular process in circulatory system	4	2.05E–03
GO:0048878 chemical homeostasis	8	6.64E–03
GO:0015698 inorganic anion transport	4	7.74E–03
GO:0008015 blood circulation	5	8.74E–03
GO:0003013 circulatory system process	5	8.74E–03
GO:0006939 smooth muscle contraction	3	9.05E–03
GO:0045860 positive regulation of protein kinase activity	5	1.61E–02

DEG: differentially expressed genes

On the other hand, the down-regulated DEGs were primarily enriched in MOXBCP and DGM pathways, in which three genes of *GSTM1*, *GSTM3,* and *ADH1B* were all enriched (Table 2). Additionally, function of the down-regulated DEGs were significantly associated with regulation of tube size, regulation of blood vessel size, vascular process in circulatory system, chemical homeostasis, and inorganic anion transport (Table 3). Moreover, combined with the interactions of miRNA–mRNA, function of miRNAs was inferred according to that of mRNAs. As a result, the functions of miRNAs of seven targets were enriched. *PDE8B* (targeted by hsa-miR-128) was significantly enriched in cyclic nucleotide catabolic process and nucleoside monophosphate catabolic process; *SLC26A7* (targeted by hsa-miR-132 and hsa-miR-223), *SCN2A* (targeted by hsa-miR-132, hsa-miR-194, hsa-miR-195, hsa-miR-223, hsa-miR-30e, and hsa-miR-374b), and *SLC4A11* (targeted by hsa-miR-28-5p) were enriched in transport related function; *KIT* (targeted by hsa-miR-152) was significantly enriched in positive regulation of protein kinase activity, *PPP1R3C* (targeted by hsa-miR-374b) was significantly enriched in regulation of glycogen catabolic process; and *PLN* (targeted by hsa-miR-374b) were significantly enriched in homeostasis related processes (data not shown).

### PPI networks of DEGs

**Figure 1.**
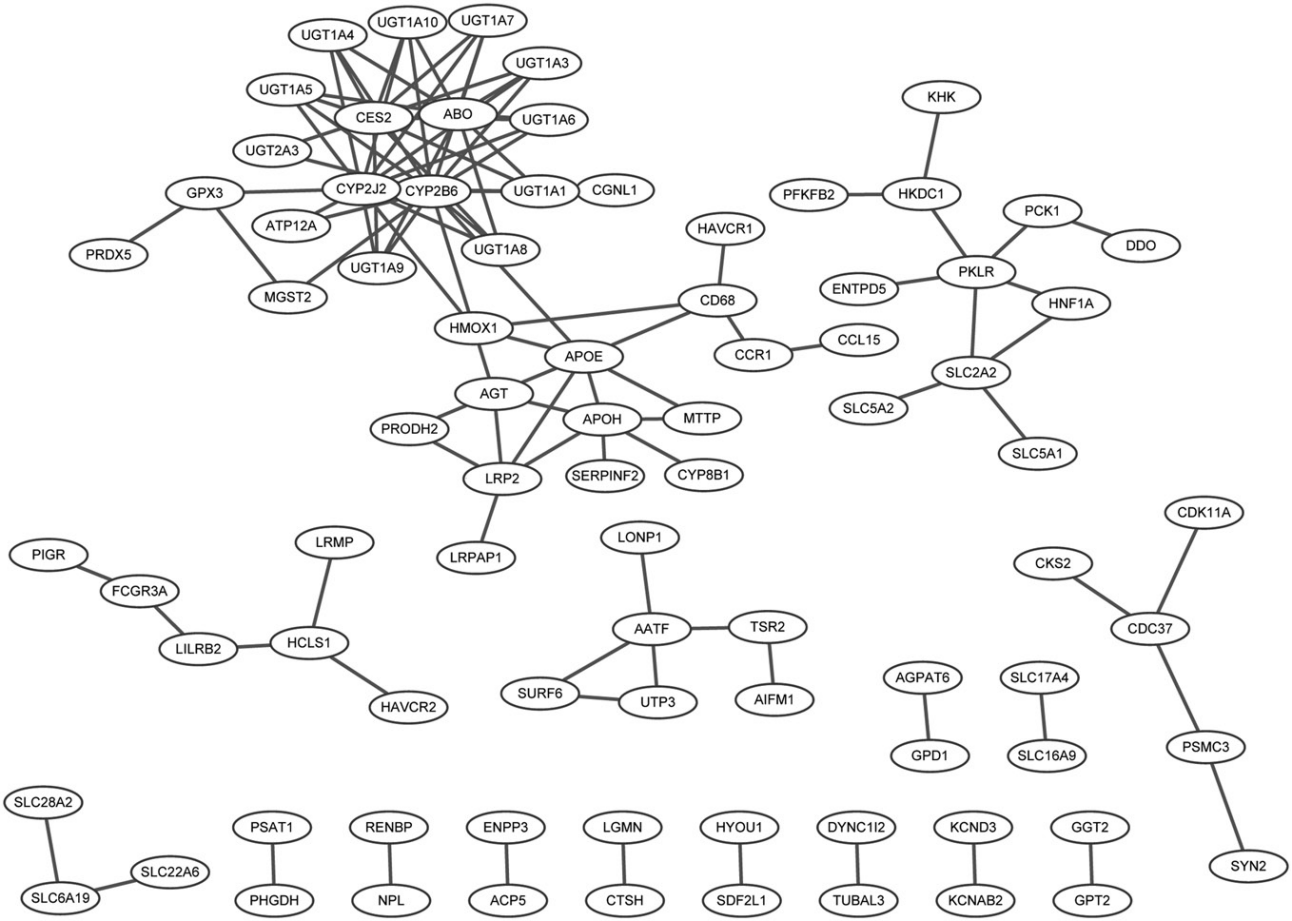
Protein–protein interaction network for up- and down-regulated differently expressed genes.

Based on information of the String database, the PPI networks of up- and down-regulated DEGs were constructed, respectively. In the PPI network for up-regulated DEGs, there were 84 nodes and 106 interactions (Figure 1), and the hub nodes (degrees >5) were *CYP2B6* (15), *CYP2J2* (12), *CES2* (10), *ABO* (9), *APOE* (7), and *APOH* (6).

For down-regulated DEGs, the integrated network of miRNAs and DEGs was established, combining with the interactions of miRNA-mRNA. In this integrated network, there were 49 nodes and 54 interactions, and *ASPN*, *COL12A1* and *SCN2A* were three hub nodes. In addition, key miRNAs like hsa-miR-30e, hsa-miR-374b, hsa-miR-128, hsa-miR-195, hsa-miR-132, and hsa-miR-223 were identified.

## Discussion

**Figure 2.**
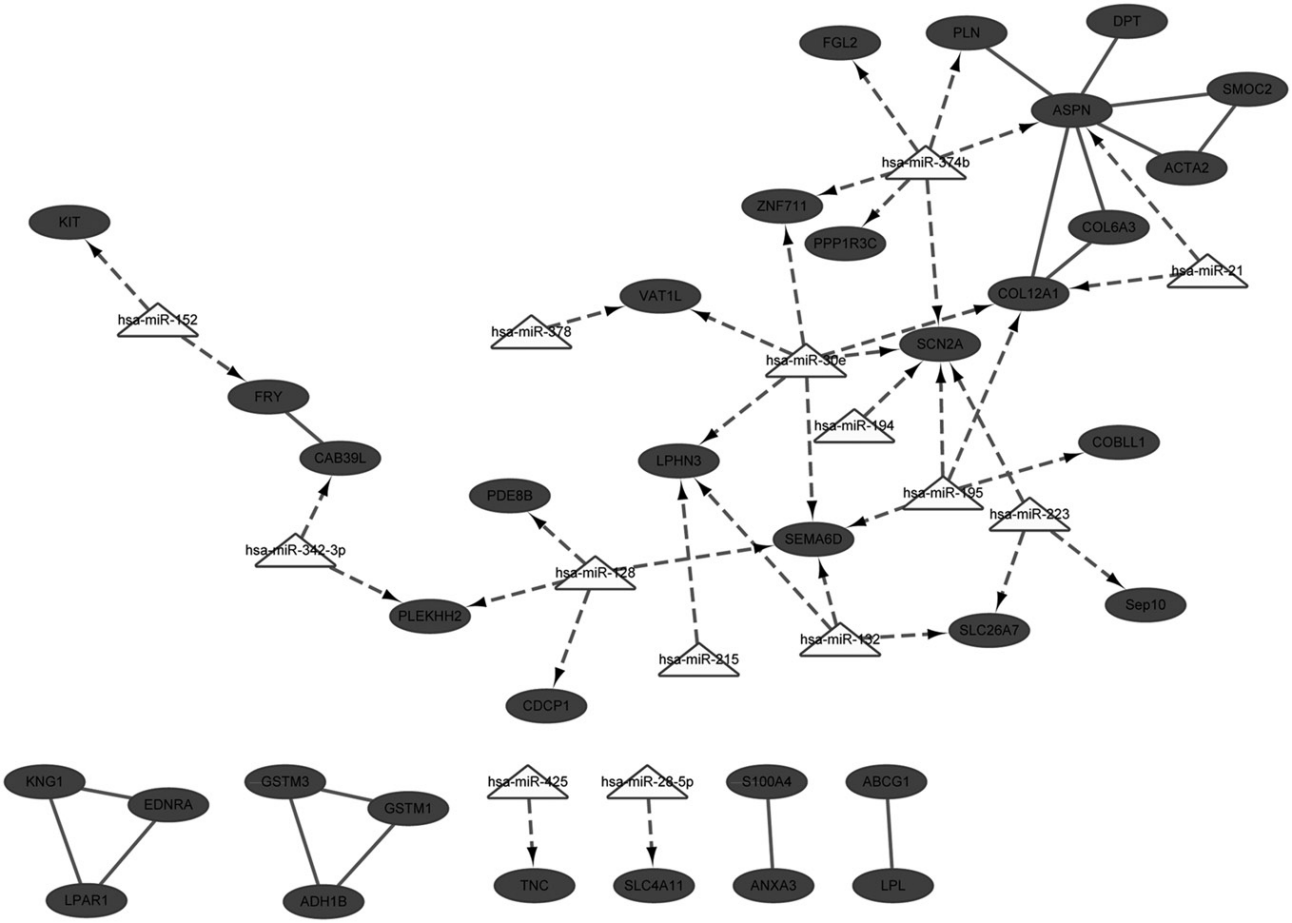
Integrated network for down-regulated differently expressed genes.

Although hypertension is a chronic disease, it is an important factor for cardiovascular disease and a major cause for mortality.^24^ In the present study, based on the analyses of microarray data, three down-regulated DEGs, *ASPN*, *COL12A1,* and *SCN2A*, were identified and predicted as targets of multiple miRNAs. As revealed in the integrated network (Figure 2), *ASPN* was the target of miRNA-374b and miRNA-21, and *COL12A1* was the target of miRNA-30e, miRNA-21, and miRNA-195, while *SCN2A* was the target of miRNA-30e, miRNA-374b, and miRNA-195. Notably, *ASPN* and *COL12A1* were linked with each other in the integrated network.

*COL12A1* is a homortrimer associated with type I collagen and its encoded protein belongs to the fibril-associated collagens with interrupted triple helices collagen family.^25^ Reportedly, in renal cell carcinoma, expression of collagen genes are associated with poor prognosis in several tumor types and *COL12A1* has been detected to be highly expressed in subtype three tumors.^26^*COL12A1* is identified as the only collagen gene that up-regulated almost two fold in the *db*/*db* diabetic nephropathy mouse model, compared with normal mice.^27^ Moreover, a recent study reveals that *COL12A1* is up-regulated in salt-induced hypertensive rat.^28^ As a class II secreted leucine-rich proteoglycans (SLRP), *ASPN* encodes a cartilage extracellular protein that could bind collagen and calcium.^29^ In response to venous hypertension, *ASPN* is found to be significantly downregulated in varicose saphenous veins, compared with normal saphenous veins (NSV) (expression value: 40.0 versus 207.1).^30^ However, most studies demonstrate *ASPN* is associated with osteoarthritis.^31^^,^^32^*ASPN* was firstly implicated in renal cortex of hypertensive patients based on our study, suggesting it might be a novel gene marker for hypertensive kidney prognosis. Both of *ASPN* and *COL12A1* are extracellular matrix (ECM) genes. Although there are no reports about their correlations, they are simultaneously down-regulated in mouse models and patient cells.^33^ In addition, *COL6A3*, one family member of *COL12A1*, is also linked to *ASPN* in the sub-networks in early phase of Duchenne muscular dystrophy, based on the expression profile of GSE6011.^34^ These collectively provide potent evidence that *ASPN* might function synergistically with *COL12A1*, as predicted in our integrated network (Figure 2).

Regarding to the targeting interactions with miRNA, *COL12A1* is reported as the target of miR-300-5p in adult rat cardiac fibroblasts^35^ and the target of hsa-miR-16 in human obesity.^36^ In the present study, *COL12A1* was predicted as the target of hsa-miR-21, hsa-miR-30e, and hsa-miR-195. Reportedly, miR-21 is one of the miRNAs that induced by Angiotensin II. It is enriched in cardiac fibroblasts and could promote cardiac fibrosis via increasing expression of MMP2 in fibroblast.^35^ Commonly, aberration of miR-21 links to development of various cancer types, and involves in numerous cellular process, such as proliferation, apoptosis and metastasis.^37^^,^^38^ In addition, *COL12A1* is identified as one novel target of miR-26b in carcinoma-associated fibroblasts of breast cancer.^39^ However, no targeting relationships between *COL12A1* and miR-21, and miR-195 are reported yet. Interestingly, in renal mesangial cells, *COL12A1* is suggested as a potential target of miR-30e*^40^ further supporting our prediction of the targeting of hsa-miR-30e and *COL12A1*. For the targeting relationships between *ASPN* and miRNAs, *ASPN* is predicted as the target of miR-21 in hippocampus after traumatic brain injury.^41^ Considering that *COL12A1* was also predicted as the target of miR-21 but no validation and other prediction have been reported, it might be inferred the targeting of miR-21 on *COL12A1* might be via the mediation of *ASPN*. However, more validation experiments are required.

Deletion of angiotensin II type 1 receptors could result in the disruption of epithelial sodium transport in proximal tubule, and then cause blood pressure alteration.^14^ The sodium channels, voltage-gated alpha subunit gene *SCN2A* is considered as one candidate for hypertension detection.^7^ However, based on current studies, this gene is mostly related to neurological disorders. Reportedly, mutations in *SCN2A* could cause seizures and epilepsy.^42^^,^^43^ With regard to the targeting miRNA and this gene, it is predicted as the target of miR-132, one of the neuronal activity-dependent miRNAs.^44^ Unfortunately, there are few studies reported potential targeting of miR-30e or miR-374b and this gene, as predicted in our study. This suggests that *SCN2A* might be a novel target of these miRNAs in renal cortex of hypertensive patients. Likewise, we need extensive experiments to validate the findings in our study.

Although we provided more regulatory information than the study of Marques, there remained a limitation that all the predictive results need to be validated via substantial experiments. Nevertheless, our study is still of great value and provide novel insight into regulatory mechanisms on hypertensive kidney.

In conclusion, our results identified three crucial genes in hypertensive kidney, such as *COL12A1*, *ASPN,* and *SCN2A*. Among them, *ASPN* was linked to *COL12A1* and they were both targets of miR-21. *SCN2A* might be the target of miR-30e and miR-374b. However, these regulatory relationships need to be further validated.

## References

[1] Cloutier L, Schiffrin EL. Hypertension prevalence and control: Impact of method of blood pressure measurement. Curr Cardiovasc Risk Rep. 2012;6:267–273.

[2] Coffman TM. Under pressure: The search for the essential mechanisms of hypertension. Nat Med. 2011;17:1402–1409. 22064430 10.1038/nm.2541

[3] Guo F, He D, Zhang W, et al Trends in prevalence, awareness, management, and control of hypertension among United States adults, 1999 to 2010. Journal of the American College of Cardiology 2012; 60:599–606. 22796254 10.1016/j.jacc.2012.04.026

[4] Büssemaker E, Hillebrand U, Hausberg M, et al Pathogenesis of hypertension: Interactions among sodium, potassium, and aldosterone. Am J Kid Dis. 2010;55:1111–1120. 20223568 10.1053/j.ajkd.2009.12.022

[5] Harris RC, Zhang M-Z. Dopamine, the kidney, and hypertension. Curr Hypertens Rep. 2012;14:138–143. 22407378 10.1007/s11906-012-0253-z PMC3742329

[6] Crowley SD, Gurley SB, Oliverio MI, et al Distinct roles for the kidney and systemic tissues in blood pressure regulation by the renin-angiotensin system. J Clin Investigat. 2005;115:1092–1099. 10.1172/JCI200523378 PMC1070417 15841186

[7] Mocci E, Concas MP, Fanciulli M, et al Microsatellites and SNPs linkage analysis in a Sardinian genetic isolate confirms several essential hypertension loci previously identified in different populations. BMC Med Genet. 2009;10:81 19715579 10.1186/1471-2350-10-81 PMC2741446

[8] Fava C, Danese E, Montagnana M, et al Serine/threonine kinase 39 is a candidate gene for primary hypertension especially in women: Results from two cohort studies in Swedes. J Hypertens. 2011;29:484–491. 21178783 10.1097/HJH.0b013e328342b2c1

[9] Clemitson J-R, Dixon RJ, Haines S, et al Genetic dissection of a blood pressure quantitative trait locus on rat chromosome 1 and gene expression analysis identifies SPON1 as a novel candidate hypertension gene. Circulat Res. 2007;100:992–999. 17332427 10.1161/01.RES.0000261961.41889.9c PMC3533402

[10] Nickel N, Kempf T, Tapken H, et al Growth differentiation factor-15 in idiopathic pulmonary arterial hypertension. Am J Respirat Crit Care Med. 2008;178:534–541. 18565955 10.1164/rccm.200802-235OC

[11] Nunez-Iglesias J, Liu C-C, Morgan TE, et al Joint genome-wide profiling of miRNA and mRNA expression in Alzheimer's disease cortex reveals altered miRNA regulation. PLoS One. 2010;5:e8898. 20126538 10.1371/journal.pone.0008898 PMC2813862

[12] Li S, Zhu J, Zhang W, et al Signature microRNA expression profile of essential hypertension and its novel link to human cytomegalovirus infection. Circulation. 2011;124:175–184. 21690488 10.1161/CIRCULATIONAHA.110.012237

[13] Jansen C, Eischeid H, Goertzen J, et al The role of miRNA-34a as a prognostic biomarker for cirrhotic patients with portal hypertension receiving TIPS. PLoS One. 2014;9:e103779. 25068403 10.1371/journal.pone.0103779 PMC4113430

[14] Marques FZ, Campain AE, Tomaszewski M, et al Gene expression profiling reveals renin mRNA overexpression in human hypertensive kidneys and a role for microRNAs. Hypertension. 2011;58:1093–1098. 22042811 10.1161/HYPERTENSIONAHA.111.180729

[15] Irizarry RA, Hobbs B, Collin F, et al Exploration, normalization, and summaries of high density oligonucleotide array probe level data. Biostatistics. 2003;4:249–264. 12925520 10.1093/biostatistics/4.2.249

[16] Smyth GK Limma: Linear models for microarray data Bioinformatics and Computational Biology Solutions Using R and Bioconductor. Springer, NY; 2005.

[17] Ritchie ME, Silver J, Oshlack A, et al A comparison of background correction methods for two-colour microarrays. Bioinformatics. 2007;23:2700–2707. 17720982 10.1093/bioinformatics/btm412

[18] Noerholm M, Balaj L, Limperg T, et al RNA expression patterns in serum microvesicles from patients with glioblastoma multiforme and controls. BMC Cancer. 2012;12:22. 22251860 10.1186/1471-2407-12-22 PMC3329625

[19] Kertesz M, Iovino N, Unnerstall U, et al The role of site accessibility in microRNA target recognition. Nat Genet.2007;39:1278–1284. 17893677 10.1038/ng2135

[20] Grün D, Wang Y-L, Langenberger D, et al microRNA target predictions across seven Drosophila species and comparison to mammalian targets. PLoS Comput Biol. 2005;1:e13. 16103902 10.1371/journal.pcbi.0010013 PMC1183519

[21] Da Wei Huang BTS, Lempicki RA. Systematic and integrative analysis of large gene lists using DAVID bioinformatics resources. Nat Protocols. 2008;4:44–57. 10.1038/nprot.2008.211 19131956

[22] Franceschini A, Szklarczyk D, Frankild S, et al STRING v9. 1: Protein–protein interaction networks, with increased coverage and integration. Nucl Acids Res. 2013;41:D808–D815. 23203871 10.1093/nar/gks1094 PMC3531103

[23] Smoot ME, Ono K, Ruscheinski J, et al Cytoscape 2.8: New features for data integration and network visualization. Bioinformatics. 2011;27:431–432. 21149340 10.1093/bioinformatics/btq675 PMC3031041

[24] Chow CK, Teo KK, Rangarajan S, et al Prevalence, awareness, treatment, and control of hypertension in rural and urban communities in high-, middle-, and low-income countries. JAMA. 2013;310:959–968. 24002282 10.1001/jama.2013.184182

[25] Posthumus M, September AV, O'Cuinneagain D, et al The association between the COL12A1 gene and anterior cruciate ligament ruptures. Br J Sports Med. 2010;44:1160–1165. 19443461 10.1136/bjsm.2009.060756

[26] Zhao H, Ljungberg B, Grankvist K, et al Gene expression profiling predicts survival in conventional renal cell carcinoma. PLoS Med. 2005;3:e13. 16318415 10.1371/journal.pmed.0030013 PMC1298943

[27] Brunskill EW, Potter SS. Changes in the gene expression programs of renal mesangial cells during diabetic nephropathy. BMC Nephrol. 2012;13:70. 22839765 10.1186/1471-2369-13-70 PMC3416581

[28] Liu Y, Taylor NE, Lu L, et al Renal medullary microRNAs in Dahl salt-sensitive rats miR-29b regulates several collagens and related genes. Hypertension. 2010;55:974–982. 20194304 10.1161/HYPERTENSIONAHA.109.144428 PMC2862728

[29] Salmon CR, Tomazela DM, Ruiz KGS, et al Proteomic analysis of human dental cementum and alveolar bone. J Proteom. 2013;91:544–555. 10.1016/j.jprot.2013.08.016 PMC3873800 24007660

[30] Javier BB, Rahmi O, Marc L, et al Extracellular matrix remodelling in response to venous hypertension: Proteomics of human varicose veins. Cardiovasc Res. 2016;110:419–430. 27068509 10.1093/cvr/cvw075 PMC4872879

[31] Elise Duval NB, Magalie H, Ikuyo K, et al Asporin expression is highly regulated in human chondrocytes. Mol Med. 2011;17:816–823. 21528154 10.2119/molmed.2011.00052 PMC3146605

[32] Jazayeri R, Qoreishi M, Hoseinzadeh HR, et al Investigation of the asporin gene polymorphism as a risk factor for knee osteoarthritis in Iran. Am J Orthop.2013;42:313–316. 24078942

[33] Vidak S, Kubben N, Dechat T, et al Proliferation of progeria cells is enhanced by lamina-associated polypeptide 2α (LAP2α) through expression of extracellular matrix proteins. Genes Dev. 2015;29:2022–2036. 26443848 10.1101/gad.263939.115 PMC4604344

[34] Bernardini C, Censi F, Lattanzi W, et al Gene regulation networks in early phase of Duchenne muscular dystrophy. IEEE/ACM Trans Comput Biol Bioinform. 2013;10:393–400. 23929863 10.1109/TCBB.2013.24

[35] Jiang X, Ning Q, Wang J. Angiotensin II induced differentially expressed microRNAs in adult rat cardiac fibroblasts. J Physiol Sci. 2013;63:31–38. 23007623 10.1007/s12576-012-0230-y PMC10717151

[36] Li J, Zhou C, Li J, et al Global correlation analysis for microRNA and gene expression profiles in human obesity. Pathol Res Pract. 2015;211:361–368. 25701361 10.1016/j.prp.2014.11.014

[37] Si M, Zhu S, Wu H, et al miR-21-mediated tumor growth. Oncogene. 2006;26:2799–2803. 17072344 10.1038/sj.onc.1210083

[38] Rask L, Balslev E, Jørgensen S, et al High expression of miR‐21 in tumor stroma correlates with increased cancer cell proliferation in human breast cancer. APMIS. 2011;119:663–673. 21917003 10.1111/j.1600-0463.2011.02782.x

[39] Verghese ET, Drury R, Green C, et al Role of miR-26b in carcinoma-associated fibroblasts and effect on migration and invasion of breast cancer epithelial cells. The Lancet. 2014;383:S103.

[40] Castro NE, Kato M, Park JT, et al Transforming growth factor β1 (TGF-β1) enhances expression of profibrotic genes through a novel signaling cascade and microRNAs in renal mesangial cells. J Biol Chem. 2014;289:29001–29013. 25204661 10.1074/jbc.M114.600783 PMC4200256

[41] Redell JB, Zhao J, Dash PK. Altered expression of miRNA‐21 and its targets in the hippocampus after traumatic brain injury. J Neurosci Res. 2011;89:212–221. 21162128 10.1002/jnr.22539 PMC7958494

[42] Hirose S, Mohney RP, Okada M, et al The genetics of febrile seizures and related epilepsy syndromes. Brain Dev.2003;25:304–312. 12850508 10.1016/s0387-7604(03)00026-3

[43] Herlenius E, Heron SE, Grinton BE, et al SCN2A mutations and benign familial neonatal‐infantile seizures: the phenotypic spectrum. Epilepsia. 2007;48:1138–1142. 17386050 10.1111/j.1528-1167.2007.01049.x

[44] Hansen KF, Obrietan K. MicroRNA as therapeutic targets for treatment of depression. Neuropsychiatr Dis Treat. 2013;9:1011–1021. 23935365 10.2147/NDT.S34811 PMC3735337

